# Seniors don’t use Medicare.Gov: how do eligible beneficiaries obtain information about Medicare Advantage Plans in the United States?

**DOI:** 10.1186/s12913-021-06135-7

**Published:** 2021-02-15

**Authors:** Maricruz Rivera-Hernandez, Kristy L. Blackwood, Marquisele Mercedes, Kyle A. Moody

**Affiliations:** 1 Department of Health Services, Policy & Practice, Brown University School of Public Health, Box G-121-6, 121 S. Main St. 6th floor, Providence, RI 02912 USA; 2 Center for Gerontology & Healthcare Research, Brown University School of Public Health, Providence, Rhode Island (Rivera-Hernandez) USA; 3 Warren Alpert Medical School of Brown University, Providence, Rhode Island (Blackwood) USA; 4 Department of Behavioral and Social Sciences, Brown University School of Public Health, Rhode Island (Mercedes), Box G-S121-3, Providence, RI 02912 USA; 5 Communications Media at Fitchburg State University, Fitchburg, MA 01420 USA

**Keywords:** Managed care, Seniors using the internet, Insurance choice and selection, Medicare plan compare, Medicare star ratings

## Abstract

**Background:**

Managed care programs in the US are becoming a preferred alternative among low-income individuals in the US. Every year during open enrollment, seniors can enroll in Medicare Advantage (MA) or switch MA plans. However, there is very limited information about how seniors obtain information to help them make their choices. While the Centers for Medicaid and Medicare offer online resources that are designed to enable potential beneficiaries to make informed coverage decisions, there is no information as to whether seniors use these resources, and therefore whether these resources are effective compared to other information retrieval methods.

**Methods:**

The purpose of the present study was to qualitatively explore how seniors obtain information about insurance plans in MA. We conducted semi-structured interviews with 26 MA beneficiaries from Rhode Island.

**Results:**

We found that most seniors have strong preferences for obtaining information in-person regarding benefits, cost and other plan information. Some seniors relied heavily on insurance brokers or representatives, and considered the information provided to them without questioning the potential for bias. Others consulted with family and/or friends for guidance, or to compare costs and benefits. Only a few of these seniors used the available internet resources, and in fact most of them mentioned that they did not have a computer/smart device with internet capabilities. However, among those who used and appeared to be comfortable with navigating the internet, www.medicare.gov was not discussed as a useful resource for making decisions regarding health insurance.

**Conclusions:**

This study suggests that existing online medical resource usage and effects among senior citizens in the United States may need supplementing with in-person communication among influential agents.

## Background

In the United States, when seniors turn 65 and/or are eligible for Medicare, they need to make a number of choices and enrollment decisions including whether to enroll in traditional Medicare (Parts A and B), prescription drug coverage (Part D), supplemental coverage to pay for additional costs not covered by traditional Medicare, or whether they will get coverage through Medicare Advantage (Part C) instead [1]. Through the Medicare Modernization Act of 2003, Medicare not only incorporated a prescription drug benefit plan (Part D), but it also expanded the Medicare managed care program (Medicare + Choice) through the new Medicare Advantage (MA) program [2]. This brought several changes to the program, including a revised payment methodology adjusting for enrollees’ hierarchical chronic condition, lock-in restrictions and more restrictive networks of healthcare providers [3]. Other changes to the payment and benefit structure have been implemented through the Affordable Care Act in 2010. For instance, MA plans are compensated or penalized based on their quality and allowing MA plans to incorporate benefits and services that address social determinants of health for people with complex conditions [4]. These changes in the MA program bolstered enrollment, which has now reached over 22 million of beneficiaries, or 34% of all Medicare enrollees [5]. The Congressional Budget Office expects this proportion of MA enrollees will reach 47% of all beneficiaries by 2029 [5]. MA enrollment is high among racial and ethnic minority groups often because MA plans may have lower premiums and may provide more generous benefits than traditional Medicare [6]. Approximately 55% of Hispanics and 40% of African Americans are enrolled in a MA plan [7]. In addition, a large proportion of beneficiaries have low education levels and/or annual incomes of about $20,000 [8]. Prior research has shown that MA may attract healthier beneficiaries and may not meet the care preferences of beneficiaries with complex needs, which encourages high-need beneficiaries to disenroll from MA [9].

Recently the federal government has implemented a number of executive orders to improve and make MA even more flexible by including access to telehealth services, expand benefits among people with high-needs, increase MA plans in rural areas, and allowing enrollment among people with end-stage renal disease [10]. Ahead of the open enrollment period, CMS announced [11] that MA premiums, on average, are expected to decline by 11% (US$24 in 2020 vs.US$26) – the lowest in 14 years. In addition, there will be over 2000 more plans (77% increase) than in 2017. Finally, it is projected that 42% of Medicare beneficiaries will be enrolled in MA [11]. As the number of people covered by MA plans continues to grow, it is important to consider the process through which enrollees decide on plans, as well as the obstacles encountered when choosing plans and techniques employed to overcome these setbacks.

While the Centers for Medicare & Medicaid Services (CMS) provide online resources with information about plans that are designed to enable potential beneficiaries to make informed coverage decisions among marketplace options, the use of these resources can be confusing and challenging when there is such an overwhelming number of options for consideration [12–14]. This may be even more difficult among people with low education levels or who may not have access to internet/broadband, which is more common among seniors [15]. Among the information that seniors need to evaluate when choosing their MA plan, there are premiums, cost-sharing, provider network, and services covered (preventive, acute, post-acute). In 2020, MA premiums range from US $0 to $265 [16]. MA beneficiaries can receive care through: 1) Health Maintenance Organization (HMO) plans which have more restrictive networks; 2) HMO Point-of-Service (HMOPOS) plans, which may allow some out-of-network services but may have higher out-of-pocket costs; 3) Medical Savings Account (MSA) plans, which includes a special savings account but may require a separate drug plan; and 4) Preferred Provider Organization (PPO) plans. PPO plans have both network and out-of-network providers for some services and often include drug coverage as well; 5) Private Fee-for-Service (PFFS) plans allows beneficiaries to use any Medicare-approved providers; and 6) Special Needs Plans (SNPs) provide coverage to beneficiaries with specific chronic conditions, needs or dually-eligible to Medicaid [1]. Therefore, seniors may need to evaluate a number of benefits including vision, dental, hearing, transportation, fitness, in-home support and home safety while accounting for out-of-pocket costs. Finally, there is information about the overall quality (CMS five-star rating system) of the MA plans available in the county, as well as other quality domains. Therefore, decision-making is understood to involve the 1) processing technical terminology; 2) comparing alternatives and attributes; and 3) weighing options based on preferences. However, with about 28 MA plans, on average [6], to evaluate by the aforementioned process - and with the complications posed by complex online resources- seniors can find themselves in need of ways of filtering plans and simplifying their decisions. In fact, it is not unusual to hear that seniors enrolling in the Medicare program may not understand all the information or feel confident about selecting the most appropriate plan and may be confused about Medicare enrollment choices [17, 18]. A plethora of evidence from Medicare Part D suggests that making plan choices is difficult for most seniors, who often select suboptimal plans – plans that do not minimize their out-of-pocket drug costs [19–21].

Research has shown that seniors are equally as capable in their decision-making capacities as their younger counterparts [22, 23]. However, in the absence of a decline in cognitive capability, there is a tendency towards consideration of less information when making decisions. It has been noted that evaluation of less information may be indicative of having pre-existing knowledge on the topic related to the impending decision [24]. However, with an experience as age-specific as enrolling in a MA program, it is possible that there is no significant existing domain of knowledge. Therefore, the decreased use of information may manifest from an attempt to streamline this selection process. Another way in which this simplification may manifest is the utilization of alternative sources of insurance-related information when making decisions about which MA plan to enroll in. Enrollees’ social networks may play a crucial role in simplifying insurance decision making. While *CMS Plan Finder* provides information regarding plan quality ratings, benefits, co-pays and estimated out-of-pocket cost and could be extremely useful helping seniors evaluate MA plans [16], seniors may assign a different value in information coming from family members, peers and other people in their social networks [25]. It is not unusual for seniors to seek health and health care-related advice from social networks [26]. Prior research has found that when selecting a drug plan, seniors have received help from insurance agents, state counselors, family members, health professionals (doctors or pharmacists) [27]. However, there has been no research focusing on the role of social networks or other sources of information in determining decisions regarding insurance plans, including MA plans.

The purpose of this qualitative study is to explore how seniors learned and/or obtain information about MA plans and ways in which seniors select these plans. Since most of the evidence regarding decision-making and plan selection has been derived from Medicare Part D, including inconsistencies in beneficiary choices, plan stickiness due to option overload, and plan information processing [20, 21, 28, 29]. Beneficiaries may stay with their current plans or select suboptimal plans due to a lack of understanding and/or inability to process all the information regarding benefits and out-of-pocket cost of other plans available in their county [27]. Therefore, we employed a qualitative strategy to gain a genuine understanding of the factors that seniors may employ to facilitate better plan choice selection in the MA program among seniors [18]. On this study we focused on understanding how MA consumers evaluate (and whether they use) the CMS website and tools, insurance representatives, and offices, as well as the role of “informal” social networks in comparing options; ultimately, we considered how these sources may help beneficiaries make choices. We accomplished this by interviewing a group of seniors from Rhode Island enrolled in MA. 

## Methods

### Design and sample

Semi-structured interviews in English or Spanish with 26 seniors from Rhode Island were conducted from April 2018 to March 2019, by trained members of the study team (The questionnaire is available at https://journals.sagepub.com/doi/suppl/10.1177/1077558720944284). These interviews were both in person and over the phone and lasted about 30 minutes each. However, the majority of these interviews were in-person (*n* = 18) and provided written consent. Those participants who were interviewed over the phone, verbal consent was obtained and recorded at the beginning of the interview. We used snowball and purposive sampling to recruit participants. First, we sent a description of the study to different community organizations (e.g., senior centers, libraries, health centers). Then, flyers were distributed among these sites. Nearly all Senior Centers, the public library, and farm fresh Rhode Island (farmer’s market) posted or delivered flyers to potential participants. Center directors also told participants of the study and seniors who qualified were encouraged to contact Dr. Rivera-Hernandez (Principal Investigator). Eligible criteria for potential participants included: 1) enrollment in a MA plan; 2) age 65 or older; 3) could describe choices and experiences. There was a $25 gift card as an incentive for participants (these incentives were part of the Advance-CTR grant that supported data collection and analysis for this research). Interview guide, coding, and other results from these interviews have been described somewhere else [18].

The protocol for this research and project materials were approved by the Brown University institutional review board. Informed consent was obtained from each participant (oral or written). That the IRB approved the use of oral and written consent.

### Analysis

 The interviews were transcribed and entered into NVIVO (software for qualitative analysis). Members of the study team coded the interviews – line by line using a priori and emergent codes [30, 31]. We developed priori codes from the published literature about insurance choice and our interview protocol. Emergent codes were developed using inductive thematic analysis (our coding scheme can be found at Rivera-Hernandez et al. 2020 [18]). Two members of the study team coded the first three interviews separately, and results were compared/discussed until consensus was achieved. The second author of this manuscript coded the remaining 23 interviews. These authors meet regularly to discuss findings. Data analyses were done using data briefs, meeting notes, and summary of codes and themes [32]. For purposes of analysis presented here, we were interested in understanding where and how seniors obtain information about the MA program, with particular attention to CMS reporting tools and insurance providers.

## Results

Our interviewees were 65–90 years old (mean age = 74). Half of the participants were women [20]; 14 were white, eight were Hispanic (seven were Spanish speakers), three were African-American, and one was Asian. In terms of education, some participants had less than elementary school to Master’s level (14 people have 12 or fewer years of education) and the majority retire from blue-collar occupations. Eleven of these participants were born outside the United States mainland. The majority of seniors [20] have been enrolled in Medicare for several years and 13 seniors have switched plans in the past. Four major interconnected themes emerged regarding sources of information including: 1) Dominance of insurance brokers and insurance companies; 2) Mixed benefits of social networks; 3) Online sources and the internet; and 4) and Strong in-person preference.

### Dominance of insurance brokers and insurance companies

Getting information from insurance companies was the dominant source for information among the participants in our study regardless of education levels or age category. This theme was present among 22 (6 males and 16 females) of the 26 participants. For those we interviewed, insurance companies reach seniors through representatives that visit their homes, via operating a storefront, or through mailers and/or seminars offered by the insurance companies at the library, senior centers or other community organizations. One senior explained these interactions succinctly, “*I went to the seminar and that’s how I got to meet him and then after meeting with him, I had called him and made an appointment to sit down and talk to him. So, he sat down with me and explained [Medicare] … Then when I was in the process of retiring, I called him and he hooked me up with this Medicare Advantage Plan*.” Other participants mentioned that *“A guy came to the house, said this is a better plan, and it sounded better to me,” and “They knocked on my door. Talked to me and told me about the program and everything and I signed up … I didn’t even hesitate. I signed right up*.” One person that attended the senior center on a regular basis described *“This year they had something at the center at my senior center and I just went and listened because somebody was there from United Health, Humana, and Blue Cross*.”

Most seniors who interact with insurance brokers or representatives describe how they received information from different plans and help them make the right choice, as one described “*They gave me information about the different plans about which one was better for me.”* For the most part, few seniors questioned whether the insurance company might be biased or providing reliable information. One senior explained “*I asked [the insurance broker] ‘:do I have to renew every year?’ he said ‘no what happens [participant] is this: we, when there’s changes made, we contact you. Because with Medicare Advantage, you never know when they’re gonna make changes. Sometimes they make changes for the good, sometimes they make changes for the bad, but when they make changes, we always contact you in ample time*.’” When another senior was asked by an interviewer if she was ever concerned that insurance representatives were just trying to sell a plan, she answered, “*No, they don’t. All the people that I talk to, no they weren’t.”* She goes on to state *“They give you an extension number to call when they call back, and their name. That’s the only thing they do. They want you to call them back... That’s what they’ll tell you. Sign you up.”* In fact, many had developed brand associations – and even brand loyalty – over the years. When asked if name recognition was important for her, one participant answered affirmatively, explaining, “*When I heard United Health was here, so I says, “Okay, I know the company.”* When asked why s/he never considers other insurance companies one senior said, *“You know why that is, because I’ve heard that they’re not consistent.”*

While most seniors that we spoke to received their information about MA plans from insurance companies without concern, this was not always the case. One senior highlighted the challenge of relying on insurance companies for information, “*It was really disconcerting... sales is not the way that I want to make my healthcare decisions. That actually really turned me off. I was really upset about it.”* Another senior explained “*There are brokers that have favorite companies. Yeah, my boss did the same thing. We were brokers and he would get plans from all companies and then combine them and say, ‘This is the difference. This is Aetna. This is Anthem and this is Oxford. These are the costs, they’re almost the same. These are the benefits. They’re almost the same. These are the hospital and your deductible and that’s when the differences come in. ‘He would have it on a piece of paper. Then he’d say, ‘You know what. I think Anthem is better,’ because that was his thing ... With another group, he might say, ‘I think Aetna is better for you.’”* We found that even seminars offered by senior centers are run by insurance company representatives. As one interviewee mentioned, *“The senior center organized the meetings. Invites different representatives from different insurance companies … Usually, the insurance companies go through senior centers where they are offering insurance to seniors.”*

### Mixed benefits of social networks

About 11 females and 1 male, across different levels of education, reported talking to or receiving advice from family and/or friends about MA plans. For instance, a senior stated “*My wife is the one that makes the decisions. She looks for information and I have to agree with what she says. Sometimes I would like to have a different plan, but the copays are expensive*.” However, some seniors preferred to make their own choices without help from their family, as one senior highlighted when asked if she received help from family members “*No, no, sometimes they have their ideas and I have my ideas. I got three daughters … They think they’re my mother. I know they mean well, but I’m still able to do [things] for myself*.”

Similarly, some seniors reported relying heavily on the experience and advice of others. For instance, one person mentioned “*I ask my friends and we exchange information about our plans … and how is it going. I also talk about my experiences.*” Another senior  explained how social gatherings lead to discussions about plans, *“People always talk about what plan they have. You know women when we get around and there’s a lot of women, everybody tells you what their plan is, you know, so you learn from that.”* Some further described how social networks serve as sources of vetted information. For example, one woman told us about a friend who has the same plan, *“I look up to him, you know? When I have any problems I call him, you know. And he says, “[name], we stay with it. It’s a good plan.” So I go along with him, you know?”* Another senior discussed how her friend helped her with disenrolling from fee-for-service and look into the MA program.

*“Well, a friend of mine made a suggestion that I thought was good, I don't take a lot of medications and I don't use western medicine for the most part and type of things I use for my healthcare Medicare, most insurance won't pay for it. That's why I was okay with the straight, but I'm getting older and so at some point maybe I need a joint replacement or something and this friend said, ‘Well, you ought to get enrolled in something that covers the supplement because you might need something and then you're paying all this out of pocket.’ So I thought that was a good thing to think about, there are a lot of choices and ... And so for me it was good that there was a choice that didn't cost me any more 'cause that was important, I'm paying for all my holistic alternative types of treatments myself, and so I ... And I'm living on social security right now, so it was important for me not to pay more.”*

Friends are not only sources of trusted information and experience, but also embodied examples of the way health status can impact your perception of a plan; “*I would ask my friends what they have because I would choose a friend that had the same [medical] problems that I did, and say, ‘How does that work for you.’”* This connection between health plan, health status and how it relates to social networks is illustrated by a quote from another senior who avoids advising friends who have different health conditions. *“I can’t give anybody advice because I don’t know how their health is. I can’t ... I think if they want advice they go to the insurance.”* This quote also illustrates the predominance of the insurance company as a trustworthy source of information before choosing a plan. While the majority expressed positive associations with peer discussions, others feel strongly that a person’s health care (and the financial implications that are involved in such a decision) are private, and potentially taboo for conversation. One senior stated, *“I don’t talk to anybody else about my insurance plan, I keep that to myself … I mind my own business. So, I mean, I’m not going around asking. Um people you know, what? What plan do you have then? I’m not like that. I like to mind my own business. Let them do what they want, I do what I want”.*

### Online sources and the internet

Participants’ experiences with online technology was both positive and negative. Some stated outright that they do not know how to find information online. When we asked whether they have a computer, twelve participants (3 males and 9 females with various levels of education and across age groups) reported that they do not own one. For instance, a senior highlighted “*No I am computer illiterate. in fact, I still have a flip phone.*” This was not unusual, as another participant mentioned, “*I’m sorry to tell you that I have no computer … I have an old flip phone*,” while another one mentioned “*No. No. I think a lot of it is most people older like me aren’t used to using the computer for everything*.” Similarly, another senior mentioned “*You know, I can’t [use the computer], and that’s another thing too. Uh, many people do not know about the computer or want to know so they have no knowledge*.” This was the case for some participants. For instance, a participant responded “*No. I never look for information,*” when asked if she ever used the internet.

Even those that are comfortable using a computer often do not own one and emphasized that they did not use it when comparing plans, enrolling, or switching. These seniors have at least high school level education. The five (four females and one male) that did use the internet/computer tend to reference insurance company information and not the CMS website. As one senior said “*I went online to find the information. I looked at different plans. Uh Blue Cross, United Health, and I compare the plans.*” Similarly, another person mentioned that she went online but “*I didn’t go on [Plan Compare]* *Medicare.gov**. It piped in ... They gave me a different website and it gave me different companies*.” When asked what website she used, the participant answered “*I googled Medicare Insurance Plans Rhode Island … and it came up*.” Similarly, another person said “*I just went on the uh, insurance company’s website.*” Oner participant mentioned that government website may be difficult to navigate stating, “*Honestly, I find it a lot easier to find things over the internet than it is to use government websites.*” None of the seniors that we interviewed referenced the CMS Plan Compare website. Very few of those interviewed knew that the website existed, and even fewer expressed an interest in using it after finding out about the site.

### Strong in-person preference

All of the participants expressed a strong preference for getting information in-person or over the phone; “*Person-to-person, I feel more comfortable*.” In fact, when asked what would help with the choice process, a senior answered “*Hands-on and face-to-face, giving a senior a brochure is not going to do it* … *They need to talk to somebody about it or hear, have a discussion about it.*” A participant mentioned “*So, even if I need to call, I go to the [office] or building and do it there”* Another participant also mentioned that going to the insurance office was more convenient “*I’m not that far away from it, I go to it, and I don’t like waiting on the phone … It’s easier to go and see someone*.” Later she added, “*If I could get a real person all the time right away when you called, it would be better, but they try and automate it as much as possible* … *it gets annoying sometimes when you have to go through five and six layers to get somebod*y.” Another participant described a similar experience; she said “*You can go to the [office] ask questions and it’s really nice … I don’t like the phone. I’d rather talk to people... person-to-person, I feel more comfortable... this technology has gotten way out-of-hand. You need personal contact*.” The same participant later described about how her insurance decisions were the results of those interactions “*I sit with somebody and they help me make a decision, they explain everything most of the time … I stay with what I have*.”

 Participants described  representatives making house calls, brick and mortar buildings, customer service lines, and seminars. One participant summed up the process, *“I prefer to go to the Blue Cross store and ask. Talk to someone. But if I was desperate and I couldn’t get there, I would just call Blue Cross. And they are very accommodating. I think Blue Cross is great.”* One participant mentioned, “*There’s an office like a half a mile from me … met with somebody and sat down and did it.*” Another senior described *“[He] sat down with me and he went over this booklet and I had this booklet right in front of me and he was going over the plans and, uh, we figured out what the best plan would be for me.*”

## Discussion

In this qualitative study about how seniors’ process of gathering information for decisions related to Medicare, we found that this was twofold: where the information was coming from (insurance company, government, broker, friend, etc.), and what form it took (website, mailing, phone call, home visit, seminar, storefront). In those we interviewed, participants were most concerned about the form information takes, as opposed to the source and location of the information. We found that most seniors in our study have strong preferences for obtaining information in-person regarding cost and benefits. Overwhelmingly, seniors were getting their knowdlege from the insurance companies and/or insurance brokers, and considered the advice provided to them without questioning the potential for bias or priming. The heavy reliance on insurance companies may be partially due to the fact that these companies utilize a number of methods and options and locations to facilitate interactions with senior citizens, who usually value interpersonal interaction over electronic or telephonic engagement. Others consulted with family and/or friends for guidance, or to compare costs and benefits; however, getting information from friends and peers is a potentially divisive topic and taboo among some of these seniors [33, 34].

Our results may be partially related to the “digital divide” that may be negatively affecting seniors, which is a concern in Rhode Island per the implementation of the DIGIAGE program (bridge this digital divide for older adults in Rhode Island) [35]. Factors impacting potential usage of Medicare.gov by seniors include broadband internet access, comfort with navigating online health portals, and internet literacy for conversions (decisions made regarding actions online). Overall, older seniors, especially those with lower levels of education and income, appear to use technology at lower rates than younger people including smartphone or tablet ownership, internet and home broadband, and social media [36]. Only a few of these seniors used the internet, and those who did had higher levels of education, which is consistent with prior literature [34, 37]. In fact, most seniors mentioned that they did not have a computer or smart device with internet capabilities. However, among the few who used and appeared to be comfortable or literate with the internet, *CMS Plan Compare* was not mentioned or discussed. Seniors who used the internet preferred to go to the insurance companies’ websites to seek plan information. Participants were often unaware of the *CMS tool* or did not want to use government sites as they were not easy to navigate.

This result is consistent with other studies that have found lack of awareness about the CMS healthcare compare tools publicly available on Medicare.gov among seniors who look online for healthcare information [38–40]. Instead seniors may be using social media and/or consumer review websites such as *Yelp*, *Facebook*, *Google Reviews*, and Twitter [38–40]. However, none of the seniors in the present study mentioned using social media on mobile or computer devices to discuss insurance options. Beyond fitting with the potentially taboo nature of health care discussions, this also links to existing literature on the existence of potential “filter bubbles” for online users, where information may only be provided to them through a combination of interpersonal interactions and previous search histories that are catered to their existing searches [41–43].

This lack of information-seeking behavior may be also partially related to the higher levels of satisfaction that these seniors had with their current plans. Nearly all participants, 23, were extremely satisfied with their current MA plan and have no intentions of switching. However, about 13 seniors have switched plans at some point (Information and themes related to MA plan switching and stickiness among these seniors have been reported somewhere else) [18]. Researchers have found that differences in information processing about available plans may be related to switching decisions, as well as self-motivation and self-efficacy [27]. The majority of these seniors may not be currently and sufficiently motivated to explore information about alternative MA plans in the market.

Our results are partially consistent with some findings from qualitative literature regarding choice and Medicare Part D. Stults and colleagues mentioned that even among highly educated beneficiaries [44], obtaining information from a “live” person was important when selecting health insurance. However, their participants, who have higher education levels, used *CMS resources*, and family members were not considered a reliable source of advice. Similar to our findings, Jacobson and others found that most seniors in their study did not use *CMS Medicare Plan Compare*, [17] and many sought guidance from insurance brokers, friends and family. In our study, the role of insurance brokers, agents and/or family members was particularly important. These agents act as navigators for these seniors who lack technological skills or Medicare literacy to evaluate the choices, helping them to mitigate the complexity of Medicare products. However, there may be incentives for brokers to enroll beneficiaries into specific types of plans. CMS disclose the amounts that companies pay brokers (employees from companies or independent agents) to sell MA plans and encourage consumers to consider this information when making their insurance choices [45]. Generally, there is an initial compensation fee during the first year and about 50% for subsequent renewals if the beneficiary remains enrolled in the same plan or make a “like plan type” switch [45]. Most participants in our study were not concerned or did not mention compensation fees.

Our interviews were conducted pre-coronavirus 2019 (COVID-19). Policy changes, stay-at-home orders and other COVID-19 restrictions may have changed participants’ perceptions and experience with technology and social networks. Post- covid-19 research suggests that seniors may have been forced to become tech-savvy about digital devices for their healthcare, to seek information and/or entertainment, and to communicate with relatives and friends [46]. However, over 40% of Medicare beneficiaries did not have access to a computer with high-speed access internet or smart phone with access to data [47]. It is still unknown, what proportion of seniors embraced digital services and which seniors may have remained disproportionally affected [48]. 

The present study has limitations. Qualitative studies such as this are exploratory works, ones that provide greater context for social and cultural knowledge of impact. Our results provide context regarding information seeking in the MA program among networked local beneficiaries, but should not be considered generalizable to the entire population since they are qualitative studies that are focused on a centralized population of seniors. We used purpose and snowball sampling to interview people who may have had a range of experiences regarding MA plan choice and selection in Rhode Island, but these experiences may be different from other MA markets with lower or higher MA penetration in the US. In 2020, Rhode Island had 14 plans available (vs. 28 nationwide) and about 30–40% of beneficiaries were in an MA plan (vs. 36% nationally) [49]. Although the majority of the plans in Rhode Island had higher ratings (4/5 stars), plans vary in terms of premiums (ranging from $USD 0–265, with about half of plans being zero premium), benefits, and other out-of-pocket costs (copays and/or co-insurance) [16]. We did not capture information about whether the participants were receiving prescription drug coverage (MAPD). However, 90% of MA plans included prescription coverage [49]. Future studies should aim to include participants from different markets, demographic and clinical characteristics. Finally, we did not ask questions related to vision or hearing impairment and/or other disabilities, which may impact understanding and information processing regarding Medicare options [50]. 

In addition, we did not collect specific information regarding income levels, household size, geography, media literacy and technological skills. Even though Rhode Island is the smallest state in the US and residents may have better access to health services than larger states, future studies should consider including this information since it may provide additional context about how seniors in the Medicare program navigate and process relevant information about insurance and health services. Finally, future studies should consider including information about available plans (e.g., quality, network, cost, additional benefits, drug coverage, clinical and medical conditions) and how they prioritize these benefits in order to identify interventions or approaches to improve informed and optimal-decision making. Despite these limitations, our results are consistent with other studies that have used qualitative interviews among Medicare beneficiaries [17, 44]. In addition, our study is among the first one to explore decision-making and how seniors obtain information about MA plans among MA beneficiaries.

Given the importance of initial plan choice and subsequent changes [28], Medicare education and unbiased support is needed to help beneficiaries make insurance choices. While the state health insurance assistance programs (SHIP) are available in most areas, including Rhode Island, seniors in the present study did not use this resource and appeared to be unaware of it. This organization helps seniors navigate Medicare choices, providing one-on-one assistance from volunteers [51]. However, SHIP is often understaffed and need additional funding [52]. CMS have different outreach and media materials for open enrollment including television and radio ads, social media post for *Facebook* and *Twitter* [53]. In addition, anybody can visit Plan Compare at any time. However, it is unclear whether they are reaching their target audiences. This is particularly relevant due to the large proportion of MA beneficiaries with low incomes and low educational attainment [7]. As stated by multiple participants, they use insurance companies’ sites directly. Among the seniors that googled “Medicare Advantage Plans in Rhode Island,” the CMS site was not among the first ones to appear as a searched result. Therefore, CMS may consider releasing information about whether seniors are accessing and engaging with their website to determine if additional interventions are needed to improve utilization of these resources, as well as insurance literacy and computer skills among seniors.

MA enrollment trends are only expected to continue increasing over time [6]. Future research is needed to determine key elements needed to develop a decision-support tool that will enhance comfortable decision-making among seniors. Seniors in our study mentioned that government sites are difficult to navigate. If seniors are overwhelmed with the amount of information, they may not use these resources that could be extremely helpful when making important choices such as selecting health insurance. It is therefore essential to gain understanding from stakeholders about what additional resources could be available to seniors and design tools that can help adults to make informed and higher quality insurance choices in the Medicare program. Finally, it is important to explore the current and future impact of COVID-19 on these federal and state programs, as well as resources that are heavily used among seniors. Besides seniors preferring information exchanged from a live person, a few of them relied on internet and computers from the library or attended meetings to the senior center or other community organizations to obtain information and/or help about the different insurance companies. It is unclear how the lack or modification of these programs and resources will further impact older adults during open enrollment. The inability of seniors to interact with influencers and agents in a physical form means that more seniors will need to move to electronic formats to discuss their insurance changes. This may cause further friction in plan choice, which may lead to disruption within the industry.

## Conclusion

In summary, findings from our study provided information regarding sources of information that seniors use when considering enrolling and/or switching MA plans. Overall seniors preferred human-to-human interaction and did not use CMS resources. With MA enrollment going up and selection process remaining complex for many seniors, Medicare education and unbiased resources are needed for seniors to navigate the choice set and make appropriate and optimal choices.

## Abbreviations

MA: Medicare Advantage
CMS: Centers for Medicare and Medicaid Services

## References

[1] Centers for Medicare & Medicaid Services. Medicare and You 2021: The official U.S. government Medicare handbook [Internet]. CMS; 2021. Available from: https://www.medicare.gov/sites/default/files/2020-09/10050-Medicare-and-You_0.pdf

[2] Assistant Secretary for Planning and Evaluation. History of Private Plans in the Medicare Program [Internet]. ASPE. 2015 [cited 2017 Jan 3]. Available from: https://aspe.hhs.gov/report/medicare-advantage-program-2014/ii-history-private-plans-medicare-program

[3] Medicare Payment Advisory Commission. Benefit Design and Cost Sharing in Medicare Advantage Plans [Internet]. 2004 [cited 2021 Jan 12]. Available from: http://medpac.gov/docs/default-source/reports/Dec04_CostSharing.pdf.

[4] The Kaiser Family Foundation. Medicare Advantage [Internet]. KFF. 2019 [cited 2021 Jan 12]. Available from: https://www.kff.org/medicare/fact-sheet/medicare-advantage/.

[5] Freed M, Damico A, 2019. A Dozen Facts About Medicare Advantage in 2019 [Internet]. The Henry J. Kaiser Family Foundation. 2019 [cited 2019 Sep 11]. Available from: https://www.kff.org/medicare/issue-brief/a-dozen-facts-about-medicare-advantage-in-2019/

[6] Freed M, Damico A, 2019. Medicare Advantage 2020 Spotlight: First Look [Internet]. The Henry J. Kaiser Family Foundation. 2019 [cited 2020 Feb 12]. Available from: https://www.kff.org/report-section/medicare-advantage-2020-spotlight-first-look-data-note/

[7] America’s Health Insurance Plans. Medicare advantage demographics report, 2016 [internet]. 2019. Available from: https://www.ahip.org/wp-content/uploads/MA_Demographics_Report_2019.pdf

[8] Better Medicare Alliance (BMA). A fact sheet on: Medicare Advantage demographics [Internet]. 2015. Available from: http://bettermedicarealliance.org/sites/default/files/FactSheet_Demographics_V11.pdf

[9] Meyers DJ, Belanger E, Joyce N, McHugh J, Rahman M, Mor V (2019). Analysis of drivers of disenrollment and plan switching among Medicare advantage beneficiaries. JAMA Intern Med.

[10] Centers for Medicare & Medicaid Services. Trump Administration Announces Changes to Medicare Advantage and Part D to Provide Better Coverage and Increase Access for Medicare Beneficiaries [Internet]. 2020 [cited 2020 Oct 1]. Available from: https://www.cms.gov/newsroom/press-releases/trump-administration-announces-changes-medicare-advantage-and-part-d-provide-better-coverage-and

[11] CMS. Trump Administration Announces Historically Low Medicare Advantage Premiums and New Payment Model to Make Insulin Affordable Again for Seniors [Internet]. 2020 [cited 2020 Oct 1]. Available from: https://www.cms.gov/newsroom/press-releases/trump-administration-announces-historically-low-medicare-advantage-premiums-and-new-payment-model

[12] McWilliams JM, Afendulis CC, McGuire TG, Landon BE. Complex Medicare Advantage Choices May Overwhelm Seniors—Especially Those With Impaired Decision Making. Health Aff (Millwood). 2011:18. 10.1377/hlthaff.2011.0132. 10.1377/hlthaff.2011.0132 PMC3513347 21852301

[13] Kuye IO, Frank RG, McWilliams JM (2013). Cognition and take-up of subsidized drug benefits by Medicare beneficiaries. JAMA Intern Med.

[14] Chan S, Elbel B (2012). Low cognitive ability and poor skill with numbers may prevent many from enrolling in Medicare supplemental coverage. Health Aff Proj Hope.

[15] Smith A. Older Adults and Technology Use [Internet]. Pew Research Center: Internet, Science & Tech. 2014 [cited 2015 Nov 2]. Available from: http://www.pewinternet.org/2014/04/03/older-adults-and-technology-use/

[16] Centers for Medicare & Medicaid Services. Shop & Compare Plans Now with the New Medicare Plan Finder [Internet]. 2019 [cited 2020 Feb 12]. Available from: https://www.medicare.gov/blog/shop-compare-plans-now-with-the-new-medicare-plan-finder

[17] Jacobson G, Swoope C, Perry M, Slosar MC. How are Seniors Choosing and Changing Health Insurance Plans? [Internet]. 2014 [cited 2016 Dec 19]. Available from: http://kff.org/medicare/report/how-are-seniors-choosing-and-changing-health-insurance-plans/

[18] Rivera-Hernandez M, Blackwood KL, Moody KA, Trivedi AN (2020). Plan switching and stickiness in Medicare advantage: a qualitative interview with Medicare advantage beneficiaries. Med Care Res Rev MCRR.

[19] McFadden D (2006). Free markets and fettered consumers. Am Econ Rev.

[20] Hanoch Y, Miron-Shatz T, Cole H, Himmelstein M, Federman AD (2010). Choice, numeracy, and physicians-in-training performance: the case of Medicare part D. Health Psychol Off J Div Health Psychol Am Psychol Assoc.

[21] Zhou C, Zhang Y (2012). The vast majority of Medicare part D beneficiaries still Don’t choose the cheapest Plans that meet their medication needs. Health Aff (Millwood)..

[22] Fitten LJ, Lusky R, Hamann C (1990). Assessing treatment decision-making capacity in elderly nursing home residents. J Am Geriatr Soc.

[23] Stanley B, Guido J, Stanley M, Shortell D (1984). The elderly patient and informed consent. Empirical findings. JAMA.

[24] Mather M. A Review of Decision-Making Processes: Weighing the Risks and Benefits of Aging. National Academies Press (US); 2006 [cited 2020 Feb 12]. Available from: https://www.ncbi.nlm.nih.gov/books/NBK83778/

[25] Turner AM, Osterhage KP, Taylor JO, Hartzler AL, Demiris G (2018). A closer look at health information seeking by older adults and involved family and friends: design considerations for health information technologies. AMIA Annu Symp Proc.

[26] Perry BL, Pescosolido BA (2015). Social network activation: The role of health discussion partners in recovery from mental illness. Soc Sci Med 1982.

[27] Han J, Urmie J (2018). Medicare part D beneficiaries’ plan switching decisions and information processing. Med Care Res Rev MCRR..

[28] Abaluck J, Gruber J (2011). Choice inconsistencies among the elderly: evidence from plan choice in the Medicare part D program. Am Econ Rev.

[29] Bundorf MK, Szrek H (2010). Choice set size and decision-making: the case of Medicare part D prescription drug Plans. Med Decis Mak Int J Soc Med Decis Mak.

[30] Padgett D (2012). Qualitative and mixed methods in public health. Thousand oaks, Calif.

[31] Weston C, Gandell T, Beauchamp J, McAlpine L, Wiseman C, Beauchamp C (2001). Analyzing interview data: the development and evolution of a coding system. Qual Sociol.

[32] Crabtree BF, Miller WL. Doing qualitative research. Thousand Oaks, Calif.: Sage Publications; 1999.

[33] Chotto MC, Lizano F, Rivera SM, Fuentes J. Social media and elderly people: research trends. In: HCI. 2017.

[34] Lüders M, Brandtzæg PB (2017). ‘My children tell me it’s so simple’: a mixed-methods approach to understand older non-users’ perceptions of social networking sites. New Media Soc.

[35] Office of Health Aging. digiAGE- Rhode Island -Office of Healthy Aging [Internet]. 2020 [cited 2020 Oct 1]. Available from: http://oha.ri.gov/digiAGE/

[36] Anderson M, Perrin A. Technology use among seniors [Internet]. Pew Research Center: Internet, Science & Tech. 2017 [cited 2020 Sep 28]. Available from: https://www.pewresearch.org/internet/2017/05/17/technology-use-among-seniors/

[37] Pargaonkar A, Mishra W, Kadam S. A study on elderly individuals’ attitude towards ICTs. 2019;

[38] Konetzka RT, Perraillon MC (2016). Use of nursing home compare website appears limited by lack of awareness and initial mistrust of the data. Health Aff Proj Hope..

[39] Li Y, Cai X, Wang M (2019). Social media ratings of nursing homes associated with experience of care and “nursing home compare” quality measures. BMC Health Serv Res.

[40] Ranard BL, Werner RM, Antanavicius T, Schwartz HA, Smith RJ, Meisel ZF (2016). Yelp reviews of hospital care can supplement and inform traditional surveys of the patient experience of care. Health Aff (Millwood).

[41] Pariser E. Beware online “filter bubbles” [Internet]. [cited 2020 May 20]. Available from: https://www.ted.com/talks/eli_pariser_beware_online_filter_bubbles

[42] Haim M, Graefe A, Brosius H-B (2018). Burst of the filter bubble?. Digit Journal.

[43] Bruns A (2019). Are filter bubbles real?.

[44] Stults CD, Baskin AS, Bundorf MK, Tai-Seale M (2018). Patient experiences in selecting a Medicare part D prescription drug plan. J Patient Exp.

[45] Centers for Medicare & Medicaid Services. Agent Broker Compensation [Internet]. 2020 [cited 2020 Feb 13]. Available from: https://www.cms.gov/Medicare/Health-Plans/ManagedCareMarketing/AgentBroker

[46] Hackett M. Survey shows seniors are embracing technology and telehealth during pandemic [Internet]. MobiHealthNews. 2020 [cited 2021 Jan 12]. Available from: https://www.mobihealthnews.com/news/survey-shows-seniors-are-embracing-technology-and-telehealth-during-pandemic.

[47] Lam K, Lu AD, Shi Y, Covinsky KE (2020). Assessing telemedicine unreadiness among older adults in the United States during the COVID-19 pandemic. JAMA Int Med..

[48] Graham J, Kaiser Health News. Seniors who struggle with technology face telehealth challenges [Internet]. CNN. 2020 [cited 2021 Jan 12]. Available from: https://www.cnn.com/2020/07/23/health/seniors-technology-telehealth-wellness-partner/index.html.

[49] Freed M, Damico A, 2020. A Dozen Facts About Medicare Advantage in 2020 [Internet]. KFF. 2020 [cited 2020 Sep 24]. Available from: https://www.kff.org/medicare/issue-brief/a-dozen-facts-about-medicare-advantage-in-2020/

[50] Willink A, Reed NS (2020). Understanding medicare: hearing loss and health literacy. J Am Geriatr Soc..

[51] State Health Insurance Assistance Programs. Home [Internet]. State Health Insurance Assistance Programs. 2020 [cited 2020 Feb 13]. Available from: https://www.shiptacenter.org/

[52] Huffman KF, Upchurch G (2018). The health of older Americans: a primer on Medicare and a local perspective. J Am Geriatr Soc.

[53] Centers for Medicare & Medicaid Services. CMS: Medicare Open Enrollment Period Outreach & Media Materials [Internet]. 2019 [cited 2019 Dec 11]. Available from: https://www.cms.gov/Outreach-and-Education/Reach-Out/Find-tools-to-help-you-help-others/Open-Enrollment-Outreach-and-Media-Materials

